# Disulfiram and Its Derivatives: An Immortal Phoenix of Drug Repurposing

**DOI:** 10.3390/ph19020200

**Published:** 2026-01-24

**Authors:** Ziad Omran, Omeima Abdullah

**Affiliations:** 1 King Abdullah International Medical Research Center, King Saud Bin Abdelaziz University for Health Sciences, Jeddah 21423, Saudi Arabia; 2 College of Pharmacy, Umm Al-Qura University, Makkah 21955, Saudi Arabia

**Keywords:** disulfiram, dithiocarbamate, aldehyde dehydrogenase, drug repurposing

## Abstract

Disulfiram (DSF) is a well-established inhibitor of aldehyde dehydrogenases (ALDHs) and an FDA-approved drug for chronic alcoholism. DSF has gained attention as a versatile scaffold for drug repurposing. Its metabolite, diethyldithiocarbamate (DDTC), mediates multiple biological effects via metal chelation and covalent modification of key cysteine residues. Beyond its established anticancer properties, DSF modulates cancer stem cells, reactive oxygen species, proteasome function, and drug-resistance pathways. It also shows promise in metabolic disorders, including type 2 diabetes and obesity, by targeting enzymes such as fructose-1,6-bisphosphatase and α-glucosidase, and influences energy expenditure and autophagy. DSF exhibits antimicrobial and antiparasitic activity, enhances antibiotic efficacy against multidrug-resistant bacteria, and demonstrates antischistosomal and anti-Trichomonas effects, while also providing radioprotective benefits. The clinical translation of DSF is limited by poor solubility, rapid metabolism, and off-target effects; consequently, the development of DSF analogs has become a major focus. Structural optimization has yielded derivatives with improved selectivity, stability, solubility, and target specificity, enabling precise modulation of key enzymes while reducing adverse effects. A key structure-based strategy involves introducing bulkier substituents to exploit differences in ALDH active-site architecture and achieve target selectivity. This concept is exemplified by compounds (**1**) and (**2**), in which bulky substituents confer selective inhibition of ALDH1A1 while sparing ALDH2. This review provides a comprehensive overview of DSF analogs, their molecular mechanisms, and therapeutic potential, highlighting their promise as multifunctional agents for cancer, metabolic disorders, infectious diseases, and radioprotection.

## 1. Introduction

Disulfiram (DSF) is an FDA-approved drug that has been used since 1951 to treat chronic alcoholism and is generally well tolerated. Its primary mechanism of action involves the irreversible inhibition of aldehyde dehydrogenase (ALDH), resulting in the accumulation of acetaldehyde in the bloodstream. This accumulation triggers a series of highly unpleasant physiological reactions, including hypotension, tachycardia, tachypnea, vomiting, and vertigo, when individuals treated with DSF consume even small amounts of alcohol [1].

In recent years, growing evidence has highlighted the potential of repurposing DSF for the treatment of various human cancers. Numerous studies have demonstrated its antitumor effects, particularly when combined with copper (DSF/Cu), in malignancies such as breast cancer [2], liver cancer [3,4], pancreatic cancer [5], glioblastoma [6], neuroblastoma [7], and melanoma [8]. These effects occur through several mechanisms, including the induction of intracellular reactive oxygen species (ROS) [9], activation of various cell death signaling pathways [10,11], inhibition of proteasome activity [12], and suppression of nuclear factor-kappa B signaling [13]. In addition, DSF/Cu targets cancer stem cells [14], providing a novel strategy to prevent tumor recurrence and metastasis [10]. It also interferes with several molecular pathways associated with drug resistance, making it a promising approach to enhance the sensitivity of chemo-resistant and radio-resistant tumors [15]. Currently, DSF is undergoing several clinical trials for different types of cancer, such as metastatic breast cancer, glioblastoma, and recurrent pancreatic carcinoma [16].

In addition to cancer, emerging data support a potential role for DSF in the treatment of inflammatory disorders, such as such as inflammatory bowel disease [17], inflammatory injury of the kidney [18] and liver [19], osteoarthritis [20], and uveitis [21]. Its anti-inflammatory effects are linked to several mechanisms, including the inhibition of pyroptosis through covalent modification of gasdermin D [22] or inactivation of the NOD-like receptor protein 3 inflammasome [23], modulation of intracellular ROS production [23], and suppression of angiogenesis [24].

DSF also shows promising potential for treating metabolic disorders such as type 2 diabetes and obesity. Animal studies have demonstrated that DSF can reduce body weight, improve insulin sensitivity, and reverse obesity-related metabolic dysfunctions [25,26]. The underlying mechanism appears to involve reduced feeding efficiency and increased energy expenditure, potentially mediated through the activation of autophagy [27].

Building on its ability to modulate reactive oxygen species in metabolic pathways, DSF has also demonstrated promising antimicrobial activity. Although it is primarily bacteriostatic against *E. coli* and *S. aureus*, DSF significantly enhances the bactericidal activity of antibiotics such as colistin and kanamycin, both in vitro and in murine infection models. Mechanistically, this involves zinc-dependent ROS-mediated disruption of bacterial antioxidant defenses and membrane integrity [28]. Despite the fact that in vivo concentrations of DSF achieved during standard therapy are below its MIC, its synergistic activity with colistin suggests potential clinical relevance as an adjuvant therapy [28]. Recent studies have shown that DSF can also inhibit the metallo-*β*-lactamase NDM-1 both by coordinating to its zinc ions [29,30] and by carbamylating Cys208 [31]. Additionally, DSF and its metabolites can inactivate NDM-1 by oxidizing the Zn(II)-thiolate in its active site [31], thereby restoring meropenem sensitivity in multidrug-resistant strains. DSF has also been reported to inhibit the phosphoethanolamine transferase MCR-1 via zinc coordination, enhancing colistin-induced membrane damage and resensitizing clinical MCR-1-positive isolates to colistin [30]. These findings highlight the potential of DSF and its metabolites as adjuvants to potentiate existing antibiotics against resistant bacterial strains.

## 2. Molecular Mechanism of Action

**Figure 1:**
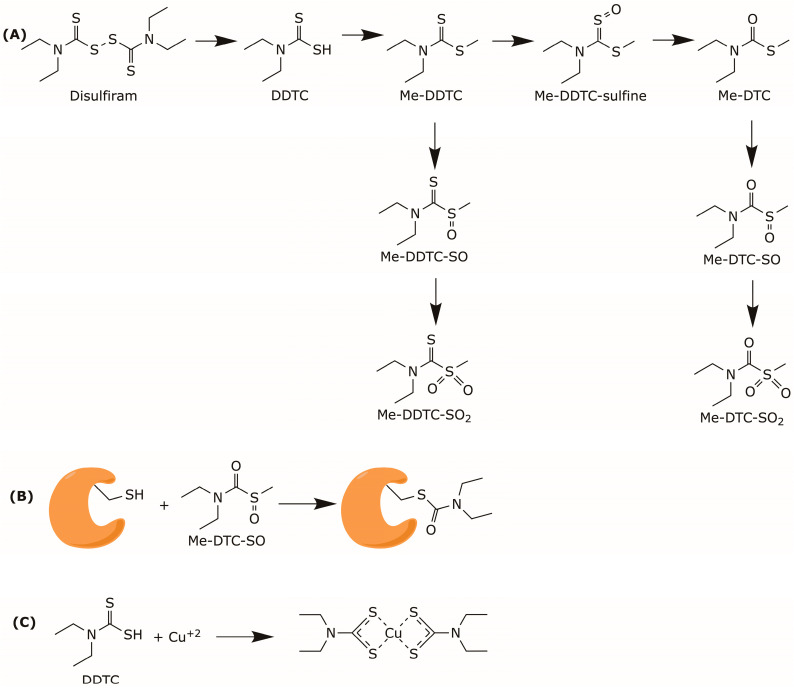
Mechanism of action of DSF. (**A**) In vivo metabolism of DSF. (**B**) Carbamoylation of cysteine containing enzymes. (**C**) Chelation of copper ion by DDTC.

Mechanistically, DSF exerts its pharmacological effects through two distinct mechanisms mediated by its reactive metabolites [32]. The first involves the irreversible carbamoylation of cysteine residues, either catalytic or located near enzymatic catalytic sites. The second is its ability to chelate metal ions, particularly copper, Figure 1.

In vivo, DSF is first reduced into diethyldithiocarbamate (DDTC), which inhibits ALDH in vivo but not in vitro [33], Figure 1A. DDTC is subsequently converted into *S*-methyl-*N*,*N*-diethyldithiocarbamate (Me-DDTC) by hepatic thiol methyltransferases. Oxidation of Me-DDTC by cytochrome P450 yields *S*-methyl-*N*,*N*-diethyldithiocarbamate-sulfoxide (Me-DDTC-SO) and *S*-methyl-*N*,*N*-diethyldithiocarbamate-sulfone (Me-DDTC-SO_2_). Additionally, Me-DDTC is also metabolized by flavin monooxygenase into *S*-methyl-*N*,*N*-diethyldithiocarbamate-sulfine (Me-DDTC-sulfine), which is further hydrolyzed into *S*-methyl-*N*,*N*-diethylthiocarbamate (Me-DTC) [34]. The latter is then oxidized by cytochrome P450 into *S*-methyl-*N*,*N*-diethylthiocarbamate-sulfoxide (Me-DTC-SO) and *S*-methyl-*N*,*N*-diethylthiocarbamate-sulfone (Me-DTC-SO_2_).

Among these metabolites, Me-DTC-SO is considered the most stable and is primarily responsible for ALDH2 inhibition, Figure 1B. In contrast, DDTC acts as a potent chelator of transition divalent metal ions, particularly copper [35]. By sequestering essential cofactors, DDTC can disrupt the activity of metal-dependent enzymes such as ALDH [16], Figure 1C.

## 3. Pharmacokinetics:

DSF is efficiently absorbed after oral dosing, with an estimated bioavailability of 80 to 90 percent. Under the acidic conditions of the stomach, disulfiram is converted into the highly hydrophobic Cu(DDTC)_2_, which facilitates its absorption across the upper gastrointestinal tract. The high lipid solubility of DSF and its metabolites facilitates their widespread distribution into adipose tissues throughout the body, while their ability to cross the blood–brain barrier enhances distribution within the central nervous system. Human studies indicate half-lives of approximately 7 h for disulfiram and 15 h for DDTC, reflecting substantial inter-subject variability in systemic levels of the drug and its metabolites. Disulfiram metabolites are eliminated through renal, fecal, and pulmonary routes. Renal excretion occurs primarily as glucuronide conjugates of DDTC or as inorganic sulfate, whereas pulmonary elimination involves the release of carbon disulfide. In addition, approximately 20 percent of the intact drug is excreted unchanged in the feces [36,37,38].

Lee et al. reported that the maximum plasma concentration of disulfiram achieved in humans after oral administration of 500 mg, the highest recommended clinical dose, was approximately 0.34 μM [39]. This level is markedly lower than the concentrations commonly used in in vitro experiments, suggesting a discrepancy between experimentally effective concentrations and clinically achievable systemic exposure [32]. This disparity may help explain, at least in part, why many mechanistic effects of disulfiram have not translated into approved therapeutic applications. Collectively, these observations highlight the need for advanced delivery approaches, optimized disulfiram analogs, or prodrug strategies to improve pharmacokinetic performance and enhance translational potential.

In this context, multiple nanodelivery strategies have been developed for DSF. Lipid-based nanoparticles have been shown to enhance systemic availability while reducing toxicity through controlled biodistribution and release. Additionally, other nanoplatforms, such as polymeric nanoparticles, nanofibers, magnetic nanoparticles, quantum dots, and metal–organic frameworks, have been explored to deliver DSF. These systems enable DSF loading via physical adsorption or chemical conjugation and allow controlled drug release in vivo [40,41].

## 4. Disulfiram Derivatives

Because of its mode of action, DSF can interfere with a wide range of enzymes and physiological pathways [32]. This promiscuity contributes to its diverse side effects, which range from mild to potentially life-threatening, the most notable being the disulfiram–ethanol reaction. Successful treatment requires strict abstinence from alcohol and avoidance of numerous household products containing alcohol, which can be challenging or impractical for many patients. Other adverse effects include hepatotoxicity, peripheral neuropathy, optic neuritis, and psychosis. In addition, disulfiram can increase the toxicity of other drugs by inhibiting cytochrome P450 reductase [16]. Therefore, the development of more selective DSF analogs is needed to achieve specific target modulation while minimizing adverse effects. This section summarizes the DSF analogs reported in the literature and their associated molecular targets.

### 4.1. ALDH1A1 Inhibitors

A growing body of evidence identifies aldehyde dehydrogenase 1A1 (ALDH1A1) as a key enzyme implicated in multiple pathological conditions. In cancer, its overexpression correlates with poor prognosis, tumor aggressiveness, and drug resistance [42], whereas ALDH1A1 inhibition depletes cancer stem cells and enhances chemosensitivity [43]. In metabolic disorders, ALDH1A1 plays a central role in adipogenesis and glucose metabolism [44,45]; its inhibition reduces body weight and improves insulin sensitivity in animal models [44,46]. In inflammatory diseases, elevated ALDH1A1 expression in intestinal macrophages contributes to the inflammatory phenotype observed in Crohn’s disease [47]. Additionally, ALDH1A1-deficient mice are viable and exhibit no apparent growth or survival defects, supporting the safety of ALDH1A1 inhibition as a therapeutic strategy [48]. Collectively, these findings support ALDH1A1 inhibition as a promising therapeutic strategy for cancer, obesity, diabetes, and inflammation [49]. Therefore, the development of DSF analogs that selectively inhibit ALDH1A1 without affecting ALDH2 is of particular clinical interest, as such compounds could provide therapeutic benefits while minimizing the adverse effects associated with ALDH2 inhibition.

**Figure 2:**
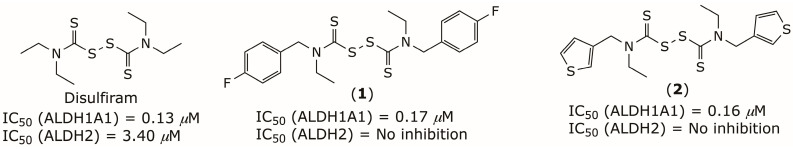
Chemical structures of disulfiram and its analogues (**1**) and (**2**), and their half-maximal inhibitory concentration (IC_50_) against ALDH1A1 and ALDH2.

Given that ALDH2 has a narrower substrate tunnel compared to ALDH1A1 [50], bulkier disulfiram derivatives are expected to selectively inhibit ALDH1A1 without affecting ALDH2 activity. Our group has developed series of disulfiram analogs in which two ethyl groups of DSF were replaced by (hetero)aromatic rings, such as *p*-fluorobenzyl (**1**) and 3-thienyl (**2**) derivatives (Figure 2) [51,52,53]. Both compounds exhibited ALDH1A1 inhibition comparable to that of DSF yet were completely devoid of anti-ALDH2 activity [54,55].

### 4.2. Monoacylglycerol Lipase (MAGL) Inhibitors

MAGL is a serine hydrolase that hydrolyzes monoglycerides into glycerol and free fatty acids [56]. Among its substrates, 2-arachidonoyl glycerol (2-AG) is the most pharmacologically relevant, as it acts as a major endocannabinoid activating both CB1 and CB2 receptors [57], thereby influencing pain sensation [58], addiction [59], neuroprotection [60], and even food intake [61]. MAGL converts 2-AG into arachidonic acid (AA), a precursor of proinflammatory prostaglandins; thus, MAGL inhibition enhances endocannabinoid signaling and reduces eicosanoid synthesis [62]. Inhibition of MAGL has also been linked to beneficial metabolic effects, such as reduced body weight, lower lipid levels [63], and altered insulin secretion [64]. Moreover, MAGL is overexpressed in several aggressive cancers [65], including prostate [66], colorectal [67], liver [68], and nasopharyngeal [69] carcinomas. Taken together, the available evidence supports MAGL inhibition as a promising therapeutic strategy for treating inflammation, neurodegenerative and metabolic disorders, and cancer.

**Figure 3:**
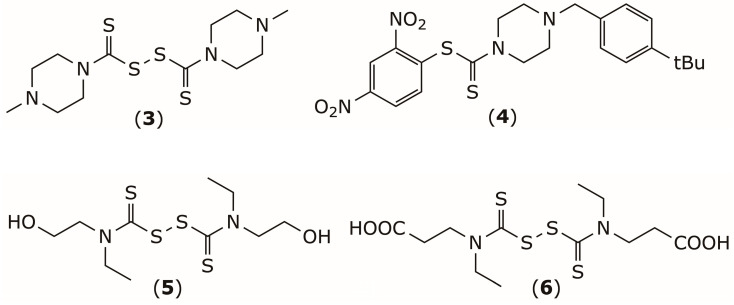
Chemical structure of MAGL inhibitors **3**–**6**.

In this context, disulfiram (DSF) has been described as a potent irreversible MAGL inhibitor (IC_50_ = 1.2 μM), acting through carbamoylation of Cys208 and Cys242 [70], which are located near the enzyme’s active site [71]. Kapanda et al. modified DSF structure to yield compound (**3**) by replacing the two ethyl group by 4-methyl-1-piperazinyl groups with IC_50_ of 0.11 μM (Figure 3) [72]. Further pharmacomodulation by the same group, involving the introduction of a 2,4-dinitrophenyl moiety, produced compound (**4**) (IC_50_ = 0.16 μM), which was capable of interacting with the catalytic Ser122 residue in addition to Cys208 and Cys242 [73]. Our group also found that introducing hydrophilic substituents into the DSF scaffold, such as hydroxyl (compound **5**) or carboxyl (compound **6**) groups, slightly enhanced MAGL inhibition, with IC_50_ values of 0.72 μM and 0.78 μM, respectively (Figure 3) [74].

### 4.3. DSF Derivatives with Antimicrobial Activity

**Figure 4:**
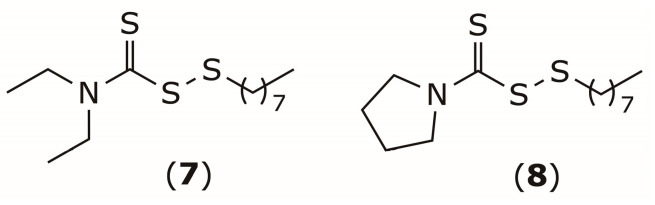
Chemical structure disulfide derivatives **7** and **8**.

DSF is a fast-acting bacteriostatic agent with a narrow antibacterial spectrum restricted to Gram-positive bacteria. It inhibits the in vitro growth of methicillin-resistant *Staphylococcus aureus* (MRSA) in a time- and dose-dependent manner, with a minimum inhibitory concentration (MIC) of 16 μg/mL [75]. DSF suppresses *S. aureus* growth by disrupting central glucose catabolism and inducing redox imbalance through oxidative stress. Additionally, its metabolite DDTC chelates metal ions and antagonizes the bacterial respiratory chain, leading to metabolic perturbations that further impair cell replication [76]. To enhance the antibacterial potency and metabolic stability of DSF, Long et al. replaced a DDTC moiety by *S*-alkylthio groups. The resulting *n*-octyl disulfide derivatives (**7**) and (**8**) (Figure 4) exhibited higher activity against MRSA, reduced cytotoxicity toward human hepatocytes, and an extended metabolic half-life [77].

**Figure 5:**
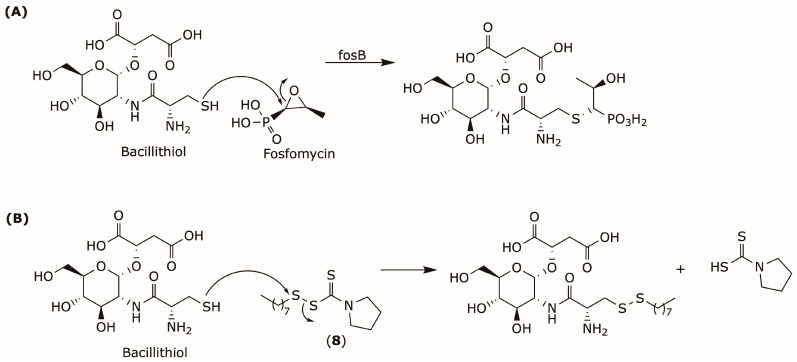
(**A**) Mechanism of fosfomycin inactivation by bacillithiol [79], (**B**) Mechanism of bacillithiol-disulfide exchange reaction with Compound (**8**) [78].

Furthermore, DSF and its *S*-octyl derivative sensitize *S. aureus* to the bactericidal effects of fosfomycin [78]. Mechanistic studies revealed that these compounds lower intracellular levels of the fosB cofactor bacillithiol via a thiol–disulfide exchange reaction, thereby depleting the cellular bacillithiol pool required for fosB-mediated fosfomycin inactivation, Figure 5.

**Figure 6:**
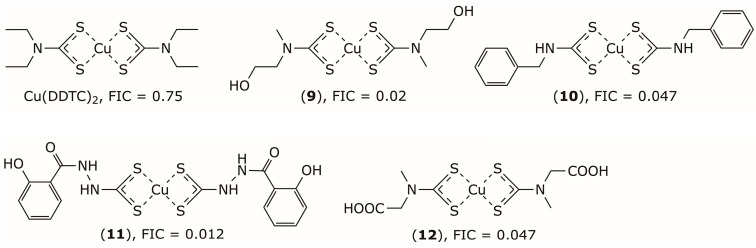
Chemical structures of Cu(DDTC)2 and its analogs (**9**–**12**) as inhibitors of NDM-1 and their FIC indices against NDM1-producing *E. coli* BL21.

DSF’s metabolite Cu(DDTC)_2_ was reported to inhibit NDM-1 by oxidizing the Zn(II)–thiolate group within its active site [31,80]. However, it did not significantly potentiate the activity of meropenem against *E. coli* expressing NDM-1, likely due to the poor ability of Cu(DDTC)_2_ to traverse the stiff outer membrane of *E. coli*. To enhance the permeability of Cu(DDTC)_2_ across the bacterial outer membrane, Yang et al. designed a focused library of dithiocarbamate consisting of four series of DDTC analogs with secondary amines (e.g., compound **9**), primary amines (e.g., compound **10**), hydrazides (e.g., compound **11**), and amino acids (e.g., compound **12**) [81], Figure 6. They investigated the structure–activity relationships and the influence of physicochemical properties on outer-membrane permeability. This screening identified compound (**9**) as a highly effective potentiator that restored meropenem activity against clinical NDM-1-producing carbapenem-resistant *Enterobacteriaceae* and slowed the development of carbapenem resistance. Notably, compound (**9**) exhibited a fractional inhibitory concentration (FIC) index of 0.02, compared to 0.75 for Cu(DDTC)_2_, highlighting its markedly superior synergistic effect with meropenem. In vivo experiments further showed that combination therapy with compound (**9**) and meropenem significantly lowered bacterial loads in the liver and spleen of a mouse infection model and effectively improved survival rates.

**Figure 7:**
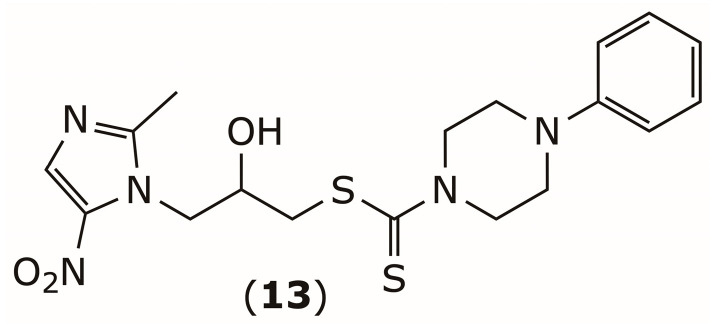
Chemical structure of the anti-*Trichomonas vaginalis* compound (**13**).

Beyond their antibacterial activity, DSF derivatives have also demonstrated potential against protozoan pathogens. Trichomoniasis, the most common non-viral sexually transmitted infection, is caused by the amitochondriate protozoan *Trichomonas vaginalis* [82]. The rising resistance to the standard drug metronidazole (MTZ) underscores the need for new therapies [83]. Mandalapu et al. developed a library of nitroimidazole–DDTC hybrid derivatives, which exhibited enhanced trichomonicidal activity in vitro against both MTZ-susceptible and MTZ-resistant strains compared to MTZ, while maintaining low cytotoxicity toward HeLa cells [84]. The most potent compound, (**13**) (Figure 7), showed minimum inhibitory concentrations (MICs) of 8.55 µM and 37.10 µM against MTZ-susceptible and MTZ-resistant strains, respectively, representing 2.4- and 9.8-fold higher potency than MTZ. This improved activity is likely due to the combined effects of the DDTC and 5-nitro groups, which synergistically inhibit hydrogenosomal function and target sulfhydryl groups in the parasite.

DSF and its derivatives have also attracted attention for their potential against helminthic infections. This is particularly relevant for schistosomiasis, a neglected tropical disease that continues to impose a major global health burden [85].

Schistosomiasis affects more than 200 million people each year and is caused by *Schistosoma* parasites. If left untreated, the disease can be fatal [86,87]. Current treatment options remain extremely limited, relying almost entirely on praziquantel [88] and, to a lesser extent, oxamniquine [89,90]. Dependence on only two drugs raises concerns about the emergence of resistance, creating an urgent need for new therapeutics [91,92]. DSF has shown notable antischistosomal activity against *Schistosoma mansoni*. In vitro, DSF produced clear effects within a short incubation period at concentrations of 25–50 μM. Treated parasites showed substantial tegumental damage, separation of worm pairs, decreased egg production, impaired motility and vitality, reduced intestinal peristalsis, and, at higher concentrations, parasite death. Male worms were particularly susceptible and displayed severe blister-like tegumental disruptions that progressed to widespread detachment and dissolution [93]. In vivo, DSF treatment reduced mortality by 60 percent in heavily infected mice and decreased granuloma formation by 80 percent, although periportal inflammation remained unchanged [94].

**Figure 8:**
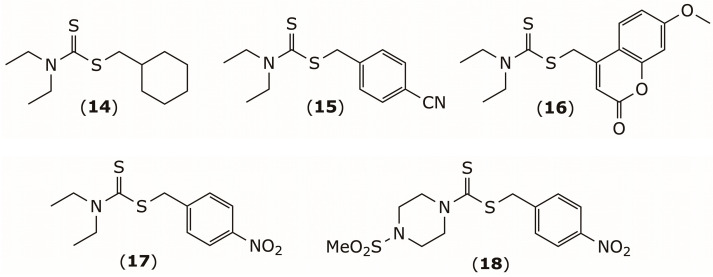
Representative structures of selected disulfiram-derived dithiocarbamate compounds with antischistosomal activity against *Schistosoma mansoni*.

Building on these observations, Rannar and colleagues developed a library of more than 100 DSF derivatives, many of which demonstrated strong in vitro activity against adult *S. mansoni* [95]. These compounds reduced egg production, impaired pairing stability, and decreased worm vitality and motility. They also caused marked tegumental damage, gut dilatation, and in some cases parasite death. Five derivatives (compounds **14**–**18**) remained active at concentrations as low as 10 μM, Figure 8. Importantly, none of the tested compounds showed cytotoxicity up to 100 μM in two human cell lines [95]. These findings collectively highlight dithiocarbamates as promising candidates for further antischistosomal drug development.

### 4.4. DSF Derivatives with Antidiabetic Activity

Consistent with the metabolic therapeutic effects of DSF highlighted in the Introduction, recent efforts have focused on its impact on specific enzymes regulating glucose homeostasis, such as fructose-1,6-bisphosphatase (FBPase). FBPase catalyzes the conversion of fructose-1,6-bisphosphate to fructose-6-phosphate and inorganic phosphate and serves as a key rate-limiting enzyme in gluconeogenesis [96]. Due to its central role in glucose production, FBPase represents an attractive drug target [97], as its inhibition can reduce glucose formation from three-carbon precursors without directly affecting glycogenolysis, glycolysis, or the tricarboxylic acid cycle [98]. Huang et al. identified cysteine residue 128 (C128) as a key allosteric site in FBPase [99]. DSF was latter shown to inhibit the enzyme’s catalytic activity by targeting C128, thereby suppressing the gluconeogenesis pathway [100].

**Figure 9:**
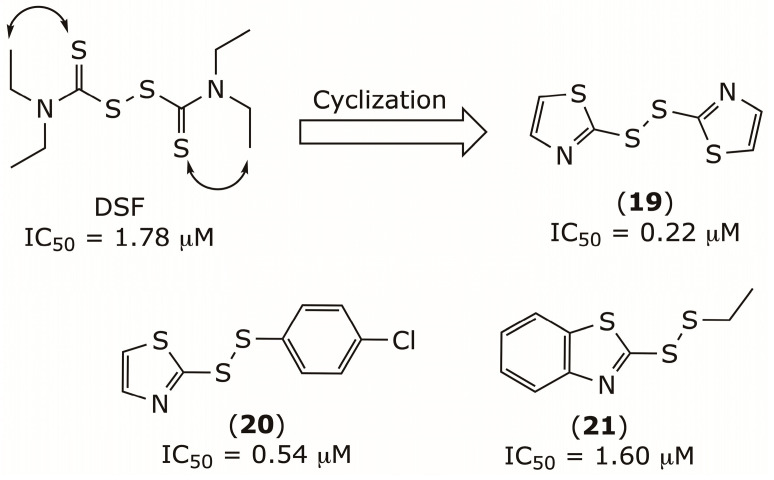
Chemical structures of FBPase inhibitors **19**–**21**.

Building on this mechanistic insight, disulfiram was structurally optimized through a cyclization strategy to produce compound (**19**) [100] together with the mixed disulfides **20** and **21** [101]. Compound **19** showed potent FBPase inhibition (IC_50_ = 0.22 μM), low cytotoxicity in HepG2 cells (IC_50_ = 75.08 μM), and a moderate hypoglycemic effect in vivo, reducing blood glucose by 54% at 25 mg/kg, Figure 9. In primary mouse hepatocytes, compound (**19**) suppressed gluconeogenesis and decreased glucose output, consistent with FBPase inhibition. In ICR mice, compound (**19**) markedly inhibited FBPase, leading to lactate accumulation, and oral glucose tolerance tests indicated moderate improvement in glucose tolerance in both ICR and diabetic *db*/*db* mice. These findings underscore the potential of compound **19** as a promising therapeutic candidate for managing type 2 diabetes.

**Figure 10:**
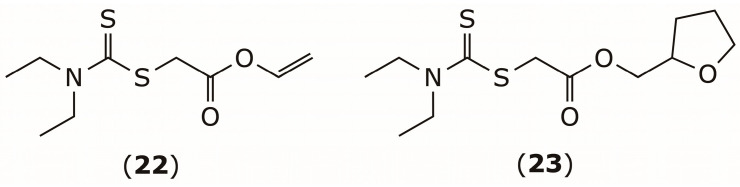
Chemical structures of α-glucosidase inhibitors **22** and **23**.

In addition to FBPase, other enzymes involved in type 2 diabetes have been explored as targets for DSF derivatives. Karimov et al. utilized the DDTC scaffold to develop ester derivatives, including compounds (**22**) and (**23**), which inhibited α-glucosidase with an IC_50_ of 28 and 56 µM, surpassing the potency of acarbose (IC_50_ = 106 µM), a standard clinical glycosidase inhibitor [102] (Figure 10). α-Glucosidase catalyzes the breakdown of complex dietary carbohydrates into simple sugars, so its inhibition slows carbohydrate digestion and absorption, helping to reduce postprandial blood-glucose spikes and offering therapeutic benefit in type 2 diabetes management [103].

### 4.5. DSF Derivatives with Anticancer Activity

**Figure 11:**
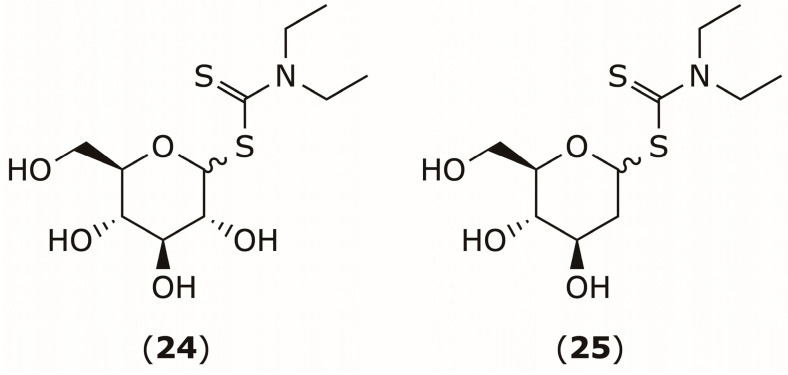
Chemical structures of DDTC prodrugs **24** and **25**.

The clinical application of DSF and DDTC as anticancer agents is limited by poor solubility and rapid plasma metabolism [104]. To address these limitations, Najlah et al. designed saccharide-linked DDTC prodrugs, such as compounds (**24**) and (**25**) (Figure 11), to improve stability, solubility, and tumor-selective activation. These prodrugs utilize thioglycosidic bonds to protect the DDTC moiety from premature degradation while preserving its metal-chelating ability to generate the active Cu(DDTC)_2_ complex. They demonstrated potent in vitro cytotoxicity, along with enhanced biostability and solubility [104].

**Figure 12:**
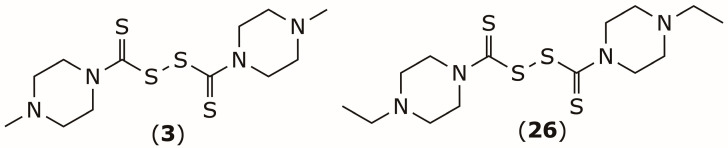
Chemical structures of DDTC piperazine derivatives **3** and **26** with radioprotective effects.

In addition to its anticancer potential, DSF has also been explored in preclinical studies for potential radioprotective effects on healthy tissues [105,106,107]. Wang and colleagues reported piperazine derivatives, compounds (**3**) and (**26**) [108] (Figure 12). When administered at a dose of 50 mg/kg, these compounds increased the 30-day survival rate by 50% and 60%, respectively, compared with vehicle-treated mice exposed to a lethal irradiation dose. These findings suggest that selected DSF derivatives may confer protection against radiation-induced damage in normal tissues and warrant further translational evaluation.

## 5. Conclusions

Disulfiram analogs are promising candidates for drug development, with improved selectivity, potency, and stability compared to the parent compound. By selectively targeting key enzymes and pathways across cancer, metabolic, inflammatory and infectious diseases, these derivatives offer novel therapeutic opportunities. Despite the substantial body of research on disulfiram and its analogs, several critical challenges continue to limit clinical translation. In particular, there remains a clear need for DSF-based agents with enhanced pharmacokinetic stability, reliable bioavailability, and predictable target engagement in vivo. Continued structure-guided optimization and translational studies are expected to advance DSF analogs toward safer and more effective clinical applications.

## Data Availability

No new data were created or analyzed in this study.
